# Insulinoma-Induced Hypoglycemia in a Patient with Insulinoma after Gastrojejunostomy for Prepyloric Ulcer

**DOI:** 10.1155/2015/127914

**Published:** 2015-10-08

**Authors:** Yavuz Savas Koca, Bünyamin Aydın, Tugba Koca, Mustafa Tevfik Bülbül, Mehmet Numan Tamer

**Affiliations:** ^1^Department of General Surgery, School of Medicine, Suleyman Demirel University, 32200 Isparta, Turkey; ^2^Division of Endocrinology and Metabolism, Department of Internal Medicine, School of Medicine, Suleyman Demirel University, 32200 Isparta, Turkey; ^3^Department of Pediatric Gastroenterology, Hepatology and Nutrition, School of Medicine, Suleyman Demirel University, 32200 Isparta, Turkey

## Abstract

Hyperinsulinism due to dumping syndrome following gastric surgery is an uncommon condition. It is specified with hypoglycemic attacks. However, linking symptoms to dumping syndrome in each patient to whom gastric surgery was performed leads to inappropriate diagnosis and therapy. Insulinoma and other causes that give rise to hyperinsulinemia should not be ignored and these diagnoses should be excluded. In this paper, 71-year-old male patient who was followed up for 2 years with a false conclusion of dumping syndrome and operated on due to insulinoma diagnosed at endoscopic ultrasonography is presented in the light of the literature.

## 1. Introduction

Hypoglycemia is a clinical syndrome that is characterized by adrenergic activation and neuroglycopenic symptoms due to the decrease in plasma glucose level. Hypoglycemia whether or not be insulin-mediated. In cases that are assumed to be healthy, the most frequent causes of hypoglycemia are drugs, insulinoma, islet cell hyperplasia/nesidioblastosis, and factitious hypoglycemia due to surreptitious administration of insulin or sulfonylureas. Though history is important for diagnosis, signs and symptoms are nonspecific. Dumping syndrome may occur in hypoglycemic patients who have a history of gastric surgery.

Hypoglycemia that is related to endogenous hyperinsulinemia is rarely seen. Pancreatic islet cell adenomas with autonomous insulin production, commonly termed as insulinoma, are rare gastropancreatic neuroendocrine tumours (NETs) with an estimated incidence of 1 or 4 per million [1].

We, herein, report a patient who underwent gastrojejunostomy for duodenal ulcer and then developed symptomatic hypoglycemia because of insulinoma.

## 2. Case Report

**Figure 1:**
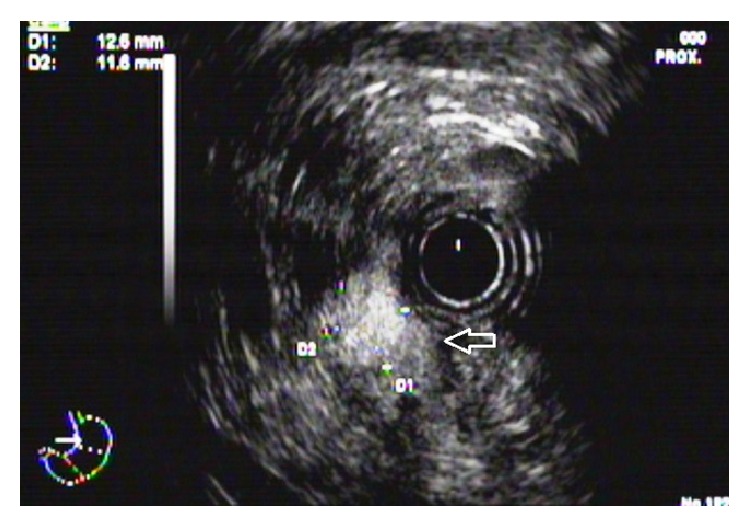
Endoscopic ultrasound image of a 12.5 × 11.6 mm hypoechoic mass located at pancreatic head.

A 71-years-old male admitted to emergency department complaining of fatigue, sweating, and unconsciousness. He had admitted to different centers for nearly 1 year with similar complaints. He had undergone antrectomy + loop gastrojejunostomy operation 2 years ago because of gastric outlet obstruction due to prepyloric gastric ulcer and shortly after the surgery, complaints started. In clinics where he had admitted with these complaints, various examinations such as computed tomography (CT) and abdominal ultrasound (US) were performed; all were in normal ranges and no pathology was observed except a minimal fall in blood glucose. The clinical diagnosis was dumping syndrome, and the patient was discharged with diet recommendations. As a result, in a clinic he admitted 1 week ago, an operation was planned for dumping syndrome. In our emergency department, blood glucose was 55 mg/dL, and he was hospitalized. Physical examination revealed that he was slightly overweight, his body mass index (BMI) was 29 kg/m^2^, blood pressure was 130/85 mm/Hg, and heart rate was 110/min. He had an operation scar from xyphoid till lower umbilicus due to previous gastric surgery. In biochemical analysis, results of complete blood count, liver function tests, and renal function tests were normal. Adrenocorticotropic hormone was 14.7 pg/mL (0–46); cortisol, 17.8 *μ*g/dL (2.32–19.52); total testosterone, 602 ng/dL; thyroid-stimulating hormone, 1.45 mIU/L (0.34–4.2); free thyroxine, 9 pg/mL (11–23); and free triiodothyronine, 2.91 pg/mL (2.5–3.9). His complaints were more severe in the morning when he wakes up, became more severe before meals with hunger, and eased with ingesting food. Before gastric surgery, he also had several deteriorations and then, it was considered as a hypoglycemic attack since he had no gastric passage. An endoscopic ultrasound was performed because the symptoms did not correlate with dumping syndrome and no pancreatic mass had been observed in pancreas by various imaging methods. Currently, a mass of 12.5 × 11.6 mm is seen in head of pancreas (Figure 1). This mass was first considered as insulinoma and a 72-hour extended fasting test was performed. In the 6th hour of the test, sweating and palpitation were observed and blood glucose was 37 mg/dL. After taking blood for C peptide and insulin, 1 mg glucagon was given subcutaneously. Blood glucose was measured at the 10th, 20th, and 30th minutes and the test was completed. Insulin was 44.6 *μ*U/Ml (*N* < 25); C-peptide, 3.6 ng/mL (0.9–7.1); and blood glucose, 55 mg/dL, 74 mg/dL, and 82 mg/dL at the 10th, 20th, and 30th minutes, respectively. A mass was extracted from pancreas with an enucleation method during operation. Histopathological examination revealed an insulinoma (well-differentiated neuroendocrine tumor). The patient was discharged at the postoperative 6th day without complication. The complaints did not recur at postoperative first and third months.

## 3. Discussion

Since signs are not specific, insulinoma can be diagnosed rather later. Clinically, symptoms of hypoglycemia belong to two groups as adrenergic (anxiety, irritability, tremor, sweating, feeling of hunger, palpitations, angina, etc.) and neuroglycopenic symptoms (dizziness, confusion, fatigue, headache, difficulty in speaking, difficulty in concentration, epilepsy, coma, temporary hemiplegia, etc.) [2]. Neuroglycopenic symptoms are observed frequently in insulinoma because extended hypoglycemia downregulates counterregulatory responses. Cases with behavioral disorders and psychiatric signs similar to our case have been reported [3]. Symptoms start several years before diagnosis; time to diagnosis is between 10 days to 20 years and patients may be followed up and treated with false diagnosis [1]. The case we present has admitted many times to different clinics and followed up with a diagnosis of dumping syndrome and could be correctly diagnosed only after 2 years.

Late dumping syndrome is a common delayed complication of bariatric surgery characterized by reactive hypoglycemia secondary to postprandial insulin surge. Symptoms such as weakness, sweating, and dizziness appear 2-3 hours after meals and occur a few months after surgery. Symptoms usually improve after a few months with dietary modifications. We moved away from diagnosis of dumping syndrome observing that the patient who had an operation before 2 years got no benefit from dietary modification.

Hyperinsulinemia with symptomatic hypoglycemia and relief of symptoms with glucose supply suggest insulinoma (Whipple triad) [1]. As in our case, hypoglycemia commonly presents in the fasting state, with the patient complaining of symptoms on waking in the morning. There is an excess weight in a quarter of cases resulting from overnutrition due to hypoglycemia symptoms [4]. Our case had a BMI of 29 kg/m^2^ and was considered overweighed. With extended (72 hours) fasting test, a biochemical diagnosis can be made in 95% of cases. Insulin (*μ*U/mL)/plasma glucose (mg/dL) (>0.27) or glucose/insulin ratio and insulin/c-peptide ratio might help in diagnosis.

Diagnosis is difficult since it is a rare condition and, also, does not have unique symptoms; symptoms are intermittent, show alterations between patients, and, more importantly, have different presentations from time to time in the same patient.

After biochemical diagnosis of insulinoma, the lesion needs to be localized. Because of high false positive and negative rates, imaging methods should not be used for diagnostic purposes. The most frequently used methods to localize pancreatic endocrine tumors are IV and oral contrast-enhanced dynamic abdominal CT. The accuracy of CT in determining primary islet cell tumors changes between 35 and 85% [5]. The aim of imaging methods in insulinoma is to determine tumor localization and evaluate metastasis. Although insulinoma has a characteristic image in CT and magnetic resonance imaging (MRI), its sensitivity is low (33–64% and 40–90%, resp.) [6]. The sensitivity of imaging methods is higher in tumors >2 cm. For the reason that tumor size was <2 cm in our case, it could not be localized with noninvasive methods.

Endoscopic US is the most sensitive test (84–93%) compared with tests such as somatostatin receptor scintigraphy, US, spiral CT, MRI, and angiography [7]. As in our case, noninvasive methods such as US and MRI are performed firstly. However, EUS can be used in cases when tumor could not be detected.

Selective arterial calcium stimulation test is an invasive dynamic test that shows tumor localization. If tumor could not be detected despite all investigations, intraoperative US and palpation are recommended [8].

Insulinoma, although rare, can lead to severe morbidity and mortality if not treated. Particularly, in cases presenting with neuroglycopenic symptoms, it should be taken into consideration in hypoglycemia etiology. The diagnosis is biochemical, but preoperative localization methods increase operation success and, also, enable less aggressive surgical procedures.
